# Systematic analysis of overall survival and interactions between tumor mutations and drug treatment

**DOI:** 10.1186/s13045-016-0249-2

**Published:** 2016-03-02

**Authors:** Francesco Gatto, Jens Nielsen

**Affiliations:** Department of Biology and Biological Engineering, Chalmers University of Technology, Göteborg, 41296 Sweden

**Keywords:** Cancer genomics, Exceptional response, Large-scale data analysis, Systems biology, Lower-grade glioma

## Abstract

**Background:**

Few exceptional responses in cancer treatment were attributed to a genetic predisposition of the tumor.

**Methods:**

We analyzed a cohort of 3105 patients from 12 different cancer types and systematically sought the existence of a correlation between overall survival and the interaction of 21 antineoplastic treatments with 6 tumor mutations.

**Results:**

We identified a single significant correlation resulting in increased overall survival from temozolomide in lower-grade glioma with *IDH1* R132H mutations. The trend could not be attributed to either the treatment or the mutation alone. Univariate and multivariate Cox regression demonstrated that this interaction stood as an independent prognostic predictor of survival.

**Conclusion:**

Our results suggest infrequent instances of exceptional responses ascribable to tumor genomics yet corroborate the existence of an interaction of temozolomide with *IDH1* mutations in lower-grade glioma.

**Electronic supplementary material:**

The online version of this article (doi:10.1186/s13045-016-0249-2) contains supplementary material, which is available to authorized users.

## Findings

The cancer genome can elicit sensitivity to certain drugs not specifically designed to target the underlying genetic aberrations. To this end, genomic markers of drug sensitivity have been systematically assessed in cancer cell lines [1, 2]. Ideally, these markers can identify patients who may better benefit from a certain antineoplastic drug [3, 4]. In contrast to the increasing availability of data about genomics of drug sensitivity in vitro [5], the association with improved patient survival is so far limited to few clinical cases, e.g., exceptional responses to everolimus in bladder cancers with *TSC1* mutations [6].

Here, we sought to systematically assess if the chances of overall survival in patients with a certain cancer type and treated with a given antineoplastic drug correlate with the presence of a certain genetic mutation in the tumor. The examined cohort comprised 3105 patients, spanning 12 cancer types (with 81–731 samples for each cancer type). Collectively, 21 antineoplastic drugs were administered each in at least 20 patients (median 82; IQR 29–150). Six cancer-associated mutations were detected in at least 20 patients in this cohort: V600E in *BRAF* (*n* = 29), R132H in *IDH1* (*n* = 108), G12V in *KRAS* (*n* = 49), H1047R in *PIK3CA* (*n* = 89), R175H in *TP53* (*n* = 45), and V777 deletion in *ZFHX3* (*n* = 22). After binning samples by cancer type, out of 1512 potential associations, 9 associations between overall survival, drug treatment, and tumor mutation had sufficient sample size for each covariate and were hereby tested. The hazard ratio (HR) for each interaction between drug treatment and tumor mutation in a cancer type was estimated in a multivariate analysis using a nested Cox proportional hazard regression model. We adopted a likelihood ratio test to test whether there is a significant effect of the interaction on overall survival on top of the tumor mutation and administered drug alone (Additional file 1: Table S1).

**Fig. 1:**
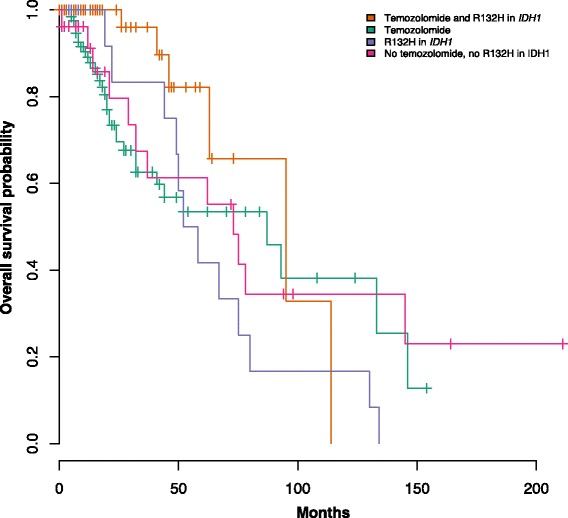
Kaplan-Meier survival plots for patients with or without an interaction between temozolomide and R132H mutations in *IDH1* in lower-grade glioma

We observed a significant effect only in one scenario, the interaction between temozolomide (TMZ) and R132H mutations in *IDH1* on the overall survival of lower-grade glioma (LGG) (likelihood ratio test *p* = 0.026). This test suggests that the correlation with survival is specific to the interaction between TMZ and R132H mutations in *IDH1* and not associated with the drug treatment or the mutation per se, as demonstrated by the Kaplan-Meier curves generated for patients stratified upon these features (log-rank test *p* = 0.047, Fig. 1). The median overall survival for patients with the interaction was 95 months (95 % CI, 63—N.E.) and for patients without the interaction was 62 months (95 % CI, 49–87).

We detected a significant prognostic value for the interaction using a univariate Cox proportional hazard regression model (*p* = 0.016, Table 1). However, the interaction violated the proportional hazard assumption and showed a time-dependent effect. The univariate analysis was also run on validated prognostic factors in LGG [7] and additional clinical features (Table 1). A multivariate analysis based on significant factors from the univariate analysis and including a time-dependent effect for the interaction revealed an independent positive correlation between the interaction and overall survival (HR 0.09, 95 % CI 0.01–0.58, *p* = 0.012), which tends to diminish over time (Additional file 1: Figure S1).

**Table 1:** Hazard ratio (HR) for clinical factors in the overall survival of lower-grade glioma

Factors	*N* [*n* death]	HR	Univariate		Multivariate		
			95 % CI	*p*	HR	95 % CI	*p*
Age	261	1.07	1.05–1.09	5e^−10^	1.07	1.05–1.09	6e^−9^
Gender							
Female	117 [30]	1					
Male	144 [33]	0.88	0.54–1.45	0.620			
Temozolomide							
No	41 [24]	1					
Yes	220 [39]	0.80	0.47–1.35	0.398			
R132H in *IDH1*							
Undetected	166 [45]	1					
Detected	95 [18]	0.74	0.43–1.29	0.292			
Interaction drug-mutation							
Absent	181 [57]	1			1		
Present	80 [6]	0.35	0.15–0.83	0.016	0.09	0.01–0.58	0.012
Histology							
Astrocytoma	107 [27]	1					
Oligoastrocytoma/oligodendroglioma	154 [36]	0.67	0.40–1.10	0.112			
Tumor grade							
Grade II	77 [18]	1			1		
Grade III	184 [45]	2.06	1.18–3.61	0.011	1.52	0.85–2.71	0.159
Laterality							
N.A.	1						
Left	131 [28]	1					
Midline	5 [1]	0.34	0.04–2.64	0.304			
Right	123 [33]	0.82	0.49–1.37	0.443			
Tumor site							
N.A./other	3				1		
Supratentorial, frontal lobe	160 [36]	1					
Supratentorial, occipital lobe	5 [1]	0.71	0.10–5.20	0.736			
Supratentorial, parietal lobe	23 [4]	0.84	0.30–2.38	0.748			
Supratentorial, temporal lobe	70 [21]	1.86	1.08–3.22	0.026	1.22	0.70–2.11	0.481
Symptoms at diagnosis							
N.A./other	19						
Headaches	62 [18]	1					
Mental status changes	22 [8]	1.84	0.80–4.27	0.153			
Motor/movement changes	22 [6]	1.20	0.47–3.06	0.698			
Seizures	119 [23]	0.58	0.31–1.08	0.087			
Sensory changes	11 [2]	1.07	0.24–4.66	0.929			
Visual changes	6 [2]	0.69	0.16–2.98	0.617			

In conclusion, we identified one genomic marker of drug sensitivity that was associated with better survival in patients, in contrast to patients treated with the same drug but with no detected mutation or vice versa. Indeed, mutations in *IDH1* were previously implicated with good prognosis in brain tumors treated with TMZ [8, 9]. Our results independently validate these findings and further extend the reach of this correlation beyond some previous limitations [10]. First and foremost, the cohort size allowed discerning that an increase in patient survival was exquisitely associated with the interaction between *IDH1* mutations and TMZ, suggestive of a synergy between treatment and tumor genomics. Second, it specifically correlated with R132H mutations. Finally, we recovered a negative time-dependent effect of the interaction, which is reminiscent of emergence of drug resistance and in line with the genetic evolution of lower-grade glioma attributed to TMZ treatment [11].

## Additional file

Additional file 1: Supplemental methods, figures, and tables. (PDF 138 kb)
