# Supplementary material for: Systematic analysis of overall survival and interactions between tumor mutations and drug treatment
Source: J Hematol Oncol. 2016 Mar 2;9:15. doi: 10.1186/s13045-016-0249-2 (PMC4774108; doi:10.1186/s13045-016-0249-2)
Supplement: Additional file 1: — Supplemental methods, figures, and tables. (PDF 138 kb) [file 13045_2016_249_MOESM1_ESM.pdf]

Title: Systematic analysis of overall survival and interactions between tumor mutations and drug treatment

Supplemental information

Supplemental Material and Methods

Data retrieval. Clinical data on overall survival and drug treatment information was retrieved for 3,105 cancer patients in the Cancer Genome Atlas (TCGA). This cohort encompasses 12 cancer types, with 81 to 731 patients for each of these 12 cancer types: invasive breast carcinoma, colon adenocarcinoma, glioblastoma multiforme, head and neck squamous cell carcinoma, kidney clear cell renal carcinoma, lower grade glioma, lung adenocarcinoma, lung squamous cell carcinoma, ovarian adenocarcinoma, pancreatic adenocarcinoma, stomach adenocarcinoma, and uterine corpus endometrial carcinoma. All patients in the cohort were administered drugs uniquely identified using ids from Drugbank (Wishart et al., 2006). Seventy-five unique antineoplastic drugs were collectively administered in this cohort. Genomic profiles for each patient tumor were obtained through the cBioPortal (Cerami et al., 2012). A list comprising 158 cancer-associated genes was queried for mutations in each profile.

Survival analysis. The associations between overall survival and presence of a tumor mutation under different drug treatments were tested using a nested Cox proportional hazard regression model within each cancer type. Our aim was to assess whether in a certain cancer type the interaction between a specific tumor mutation and the administration of a specific drug confers a different survival odds-ratio. The Cox regression model was constructed using as covariates age, the presence of a tumor mutation, the administration of a drug, and the interaction term between presence of a tumor mutation and administered drugs. We constructed a model to test for an association with the interaction only if the other covariates were observed in at least 20 samples belonging to the cancer type under examination, for example at least 20 patients were administered the same drug. The significance of the interaction between presence of a tumor mutation and an administered drug was tested using a likelihood ratio test between the Cox model with and without the interaction term. A confidence level greater than 95% was considered statistically significant. Kaplan-Meier survival curves were generated in the case of a significant interaction for each group of patients defined by the Cox model, i.e. patients with no detected mutation nor drug treatment; patients with detected mutation and no drug treatment; patients with drug treatment and no detected mutations; and patients with detected mutation and drug treatment. The significance of differential survival according to this grouping was tested using a log-rank test. A confidence level greater than 95% was considered statistically significant.

In the only instance where an interaction was deemed significant, we performed univariate analysis of overall survival using a Cox proportional hazard regression model. Relevant prognostic factors for the cancer type were retrieved within the clinical data provided by TCGA. The significance of each factor was tested individually and a confidence level greater than 95% was considered statistically significant. Hereby identified significant factors were used to construct a multivariate Cox proportional hazard regression model. The significance of each factor was tested in a multivariate fashion and a confidence level greater than 95% was considered statistically significant. The validity of the proportional hazards assumption was challenged by checking the correlation coefficient between transformed survival time and the scaled Schoenfeld residuals of each covariate. For the case in which a covariate displayed a significant non-proportional hazard, we reran the multivariate analysis by including a time-interaction variable with logarithmic effect on the covariate.

The survival analysis was implemented in R 3.1.2 using the survival and simPH packages (Gandrud, 2015).

Supplemental Tables

Table S1. Estimation of hazard ratio attributable to the interaction between a tumor mutation and administered drug in a cancer type. The statistical significance was calculated using a likelihood ratio test on a multivariate Cox regression model with or without the interaction term. NE: Not estimable.

**Cancer type**	**Tumor mutation**	**Drug**	**Likelihood ratio test *p*-value**	**Hazard ratio**	**Concordance**
Invasive breast carcinoma	H1047R in *PIK3CA*	Cyclophosphamide	0.583	NE	0.65
		Fluorouracil	1	NE	0.66
		Tamoxifen	0.752	NE	0.56
		Doxorubicin	0.077	NE	0.66
		Anastrozole	0.435	NE	0.66
		Docetaxel	0.456	NE	0.65
Low grade glioma	R132H in *IDH1*	Temozolomide	**0.024**	0.26	0.81
Pancreatic adenocarcinoma	G12V in *KRAS*	Gemcitabine	0.255	3.37	0.60
	V777 deletion in *ZFHX3*	Gemcitabine	0.457	2.07	0.63

Supplemental Figures

Figure S2. Relative hazard for the interaction between R132H mutation in *IDH1* and temozolomide in lower grade glioma over time.

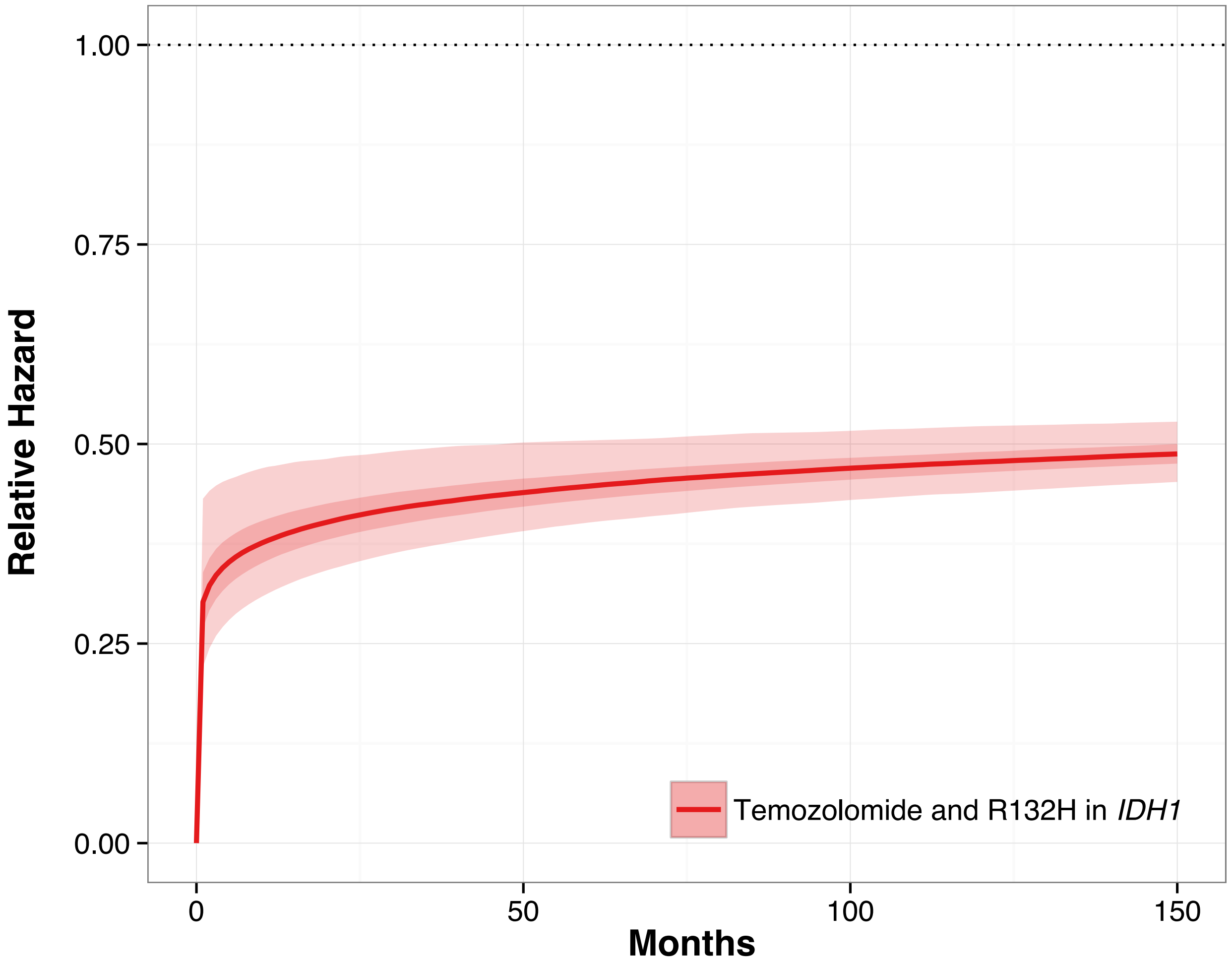

Supplemental References

1. Cerami, E., Gao, J., Dogrusoz, U., Gross, B.E., Sumer, S.O., Aksoy, B.A., et al. (2012). The cBio cancer genomics portal: an open platform for exploring multidimensional cancer genomics data. Cancer discovery 2, 401-404.
2. Gandrud, C. (2015). simPH: An R Package for Illustrating Estimates from Cox Proportional Hazard Models Including for Interactive and Nonlinear Effects. 2015 65, 20.
3. Wishart, D.S., Knox, C., Guo, A.C., Shrivastava, S., Hassanali, M., Stothard, P., et al. (2006). DrugBank: a comprehensive resource for in silico drug discovery and exploration. Nucleic acids research 34, D668-672.
